# Obstetric pain correlates with postpartum depression symptoms: a pilot prospective observational study

**DOI:** 10.1186/s12884-020-02943-7

**Published:** 2020-04-22

**Authors:** Grace Lim, Kelsea R. LaSorda, Lia M. Farrell, Ann M. McCarthy, Francesca Facco, Ajay D. Wasan

**Affiliations:** 1 Department of Anesthesiology and Perioperative Medicine, University of Pittsburgh School of Medicine, Pittsburgh, PA USA; 2 Department of Obstetrics, Gynecology & Reproductive Sciences, University of Pittsburgh School of Medicine, Pittsburgh, PA USA; 3 Magee-Womens Research Institute, Pittsburgh, PA USA; 4 UPMC Magee-Womens Hospital, 300 Halket Street, Suite 3510, Pittsburgh, PA 15213 USA; 5 The Midwife Center for Birth and Womens Health, Pittsburgh, PA USA; 6 Pittsburgh Center for Pain Medicine, University of Pittsburgh, Pittsburgh, PA USA; 7 Department of Psychiatry, University of Pittsburgh School of Medicine, Pittsburgh, PA USA

## Abstract

**Background:**

Data linking labor pain and postpartum depression are emerging. Robust, prospective evaluations of this relationship while factoring other important variables are lacking. We assessed perinatal pain and other factors predicting postpartum depression (PPD) symptoms.

**Methods:**

Third trimester women, stratified by a priori plan to receive or avoid labor epidural analgesia, were longitudinally followed from the prenatal period through labor and delivery, until 6 weeks and 3 months postpartum. Electronic pain data was collected hourly during labor in real time, capturing pain unpleasantness, intensity, pain management satisfaction, and expectations. Prenatal and postpartum data included anxiety, depression, the Brief Pain Inventory (BPI), pain catastrophizing, resiliency, and perceived social support and stress. The primary outcome was Edinburgh Postnatal Depression Score (EPDS) as a marker of PPD symptoms. The primary pain variable of interest was labor pain emotional valence (unpleasantness burden, area under the curve for entire labor duration). Single and multivariable linear regressions examined perinatal pain variables in relation to EPDS.

**Results:**

Of 72 subjects included, 55 planned/received labor epidural analgesia and 17 planned avoidance/avoided it. In the planned epidural group, the emotional valence of labor pain independently predicted six-week EPDS (labor pain unpleasantness burden, R^2^ = 0.42, *P* = 0.002). In addition to labor pain, prenatal and postpartum pain variables from the BPI independently predicted six-week EPDS. Three-month depression scores were linked to labor and acute pain (6 weeks postpartum), but not to chronic (3 months postpartum) pain variables. Intrapartum pain management satisfaction and expectations were largely met or exceeded and did not differ between analgesia groups.

**Conclusion:**

For susceptible women, pain at all perinatal time points—prenatal, labor, and postpartum—appear to be independently linked to depression scores at 6 weeks postpartum. The relationships are true, even though satisfaction and expectations regarding labor pain management were met or exceeded. These data support the concept that labor and acute postpartum pain influences both acute and long-term PPD symptoms, although additional data are needed to assess how analgesia preference interacts with these relationships.

## Background

Research has suggested a potential relationship between labor/postpartum pain and epidural labor analgesia to postpartum depression (PPD) [1–3]. Pregnant/postpartum women report a general association between pain and depressive symptoms [4–6]. One study showed referral for postpartum pain management independently predicted PPD [7]; another showed extent of epidural analgesia labor pain relief independently predicted lower PPD scores [1]. However, prospective evaluations of factors driving the labor/postpartum pain-depression relationship are limited. Further, the role of prenatal pain - which may influence both labor and postpartum pain - as an independent depression risk factor requires additional investigation. The nature of the relationship between perinatal (prenatal, labor, postpartum) pain and depression is important to understand depression’s mechanisms and modifiable risk factors.

Disentangling the relationship between perinatal pain and depression may become more complex when considering other known pain and depression risk factors. Moderator-mediator models can be used to investigate indirect associations between pain and depression. Characterizing these indirect associations is important, because identifying explanatory factors/conditions under which primary relationships change may clarify conflicting results in published literature on the relationship between parturitional pain, analgesia, and depression symptoms [1–3, 8]. Moderator-mediator analyses assess how the relationship between perinatal pain and depression changes (increases or decreases) for patient subgroups (“moderation” analysis). Mediator analyses evaluate whether perinatal pain variables are a reason for the relationship between PPD and its known risk factors, or if perinatal pain is independently associated with depression. Mediation effects clarify potential explanations for observed relationships. For example, is labor pain a mechanism for the relationship between prenatal psychological risk factors and PPD symptoms?

In this pilot prospective observational study, we assessed psychological, psychosocial, and perinatal pain factors predictive of depression symptoms. In exploratory analyses, we probed moderating-mediating effects between these factors. We hypothesized that perinatal pain variables independently predict PPD symptoms.

## Methods

The University of Pittsburgh Institutional Review Board approved the study (PRO15030338) and written informed consent was obtained from all participants. All STROBE guidelines for reporting observational studies were followed. Healthy nulliparous women, singleton gestation, in the third trimester (≥ 28 weeks) planning to use epidural labor analgesia and deliver at a hospital were included. A subgroup of women planning to avoid epidural analgesia and deliver at a community-based midwifery birthing center (The Midwife Center, Pittsburgh, Pennsylvania) were also recruited to evaluate potential differences between these populations and influence of analgesia preference on relationship between pain and depression symptoms. Investigators approached women at third trimester prenatal clinic visits; after giving written informed consent, baseline inventories were completed electronically (Research Electronic Data Capture, a secure, web-based software platform hosted at the University of Pittsburgh) within 3 days of enrollment. Women were prospectively followed; when presenting for delivery, they were deemed eligible to continue with study procedures if presenting for spontaneous or induced labor and delivery at term gestation (≥37 weeks estimated gestational age) with vertex presentation. Exclusion criteria included age < 18, non-English speaking, chronic pain history, opioid maintenance program enrollment, severe maternal obstetric disease (i.e. pre-eclampsia or eclampsia requiring magnesium therapy, necessity for immediate cesarean delivery without labor), class 3 obesity with body mass index≥40 kg/m^2^ (given its impact on failed labor epidural analgesia and data suggesting relationship between obesity and depression) [1, 9, 10], fetal anomalies or growth restriction, or contraindications to neuraxial analgesia.

### Baseline inventories

Participants electronically completed valid and reliable surveys, including baseline prenatal assessments of depression (Edinburgh Postnatal Depression Scale, EPDS) [11], anxiety (State-trait Anxiety Inventory, STAI) [12, 13], resiliency (Ego-resiliency scale, ER-89) [14], and pain catastrophizing (Pain Catastrophizing Scale, PCS) [15], and a pain inventory (Brief Pain Inventory Long Form, BPI-L) [16]. These variables were selected based on known associations with pain, depression, or both [16, 17]. Demographic characteristics were electronically self-reported and recorded, including age, race, ethnicity, estimated delivery date, as well as history of partner, sexual, domestic, childhood, substance, or other abuse, anxiety or depression, and other mental illness.

The EPDS is self-completed, 10-item scale developed specifically for women in the perinatal period. It has been shown to be an effective means of identifying patients at risk for perinatal depression [11, 18]. Importantly, the EPDS is a screening tool that is not intended to substitute for a mental health professional’s diagnosis of depression; however, the EPDS has an estimated 80% sensitivity for a diagnosis of depression [11, 18]. Because it is very sensitive, but not as specific as other instruments, it identifies almost all women who are depressed but also identifies some women who are not depressed; thus in clinical practice the EPDS is used to refer patients to a physician for evaluation based on the screen score [11, 19].

### Labor pain diary

An electronic pain diary application was programmed for this study and delivered via bedside personal device (Google Nexus 7, 2013, Android version 4.3 “Jelly Bean,” Mountain View, California). Upon birthing unit admission, the electronic diary was set up and baseline pain intensity and unpleasantness, satisfaction, and expectations were assessed. Participants were asked to rate physical and pain emotional valence hourly using a 100 mm horizontal visual analog scale: “Over the last hour, how intense has your pain been? Please mark a vertical line on the line below” and “Over the last hour, how unpleasant has your pain been? Please mark a vertical line on the line below.” Responses of 0-mm corresponded to “no intensity at all” or “no unpleasantness at all” and 100-mm indicated “the most intense pain I can imagine” or “the most unpleasant pain I can imagine,” respectively. Pain management satisfaction was assessed hourly using Pain Treatment Satisfaction Scale (PTSS) question number #55 (“Overall, how satisfied are you with your current pain management strategy?”). Participants responded using a five-point Likert scale (“1 = very satisfied,” “2 = satisfied,” “3=neither satisfied nor dissatisfied,” “4 = dissatisfied,” or “5 = very dissatisfied”). The degree that analgesia met expectations was assessed every 3 hours using PTSS question #56 (“Overall, how does your level of pain relief/management meet your expectations of pain relief/management?”) [20]. Participants responded using a five-point Likert scale (“1=greatly exceeds my expectations,” “2=somewhat exceeds my expectations,” “3=meets my expectations,” “4=does not quite meet my expectations,” or “5=does not meet my expectations at all”). Devices emitted hourly sound alerts asking participants to submit responses.

Variables recorded during and after labor included labor duration (defined as time of admission or known time of spontaneous membrane rupture until delivery), delivery mode (spontaneous vaginal, assisted vaginal, cesarean), perineal lacerations (none; first, second, third, or fourth degree), and last cervical dilation known at epidural request.

The primary pain variable of interest was labor pain emotional valence/burden, operationalized by labor pain unpleasantness burden (area under curve, AUC). AUC was calculated with the trapezoidal rule, using hourly labor pain visual analog scores until end of labor (time of delivery).

Pain diary data was used to calculate pain and analgesia scores per individual patient: post-epidural analgesia average pain (mm); labor pain intensity max (mm); labor pain unpleasantness max (mm); labor pain intensity burden (AUC); labor pain management satisfaction (average); labor pain management expectations (average). For participants undergoing epidural labor analgesia and hospital-based birth, in-hospital postpartum pain and analgesia variables were calculated based upon 0–10 numeric rating scores provided during routine clinical care: time-weighted postpartum percent improvement in pain (%) [1]; postpartum pain burden (AUC); postpartum pain max score (0–10 numeric rating).

### Labor epidural analgesia protocol

In the epidural analgesia cohort, epidural analgesia was initiated at time of patient request. Epidural space was localized by loss-of-resistance to saline and catheters were inserted at a 5 cm depth in the epidural space. Activation was by lidocaine 1.5% with 1:200,000 epinephrine (3 mL “test dose” followed by additional 2 mL), bupivacaine 0.0825% with fentanyl 2mcg/mL (8 mL), and fentanyl 100mcg. Continuous patient-controlled epidural analgesia was bupivacaine 0.0825% infusion with fentanyl 2mcg/mL 8 mL/hour, demand 8 mL, lockout of 24 mL/hour. Supplemental epidural dosing protocol included bupivacaine 0.125% 10-15 mL in 5 mL increments at first request, lidocaine 1% 10 mL in 5 mL increments with adjustment to basal infusion rate to 11 mL/hour at second request, and clinician judgement at third request.

### Postpartum day 1 or 2

Participants completed electronically recorded inventories, including pain (BPI Short Form, BPI-S), breastfeeding (yes/no), and perceived stress scale (PSSc), on the first or second postpartum day.

### Six weeks postpartum

At six weeks postpartum, participants completed electronically recorded inventories, including depression (EPDS), pain (BPI-S), and breastfeeding (yes/no).

### Three months postpartum

At three months postpartum, participants completed electronically recorded inventories, including depression (EPDS), pain (BPI-S), and breastfeeding (yes/no). Supplemental Figure 1 shows the prenatal, perinatal, and postpartum timepoints where instruments were delivered, and data were collected.

### Prenatal negative affect (NA) calculations

NA is a cluster of negative thoughts and emotions (including anxiety and depression, which involve negative thoughts and emotions) that are frequently co-morbid with pain [21–24]. High NA levels are associated with higher pain levels, poor functioning, and worse treatment outcomes [25, 26]. In this study, using validated methods described by our group, NA was calculated as a composite variable of scores of anxiety and depression, two highly comorbid conditions. Correlations between these measures are 0.60 to 0.80 in pain populations, suggesting an underlying construct. Prenatal anxiety state and EPDS scores were used to calculate three prenatal NA categories: high NA (high depression and anxiety scores), low NA (low depression and anxiety scores), and moderate NA (either high anxiety/low depression scores or high depression/low anxiety scores). NA in this model allowed assessment of a clinically important composite measure of anxiety and depression without sacrificing model variance.

### Statistical analysis

Six-week postpartum EPDS scores (continuous variable) as a measure of PPD symptoms was the primary outcome of interest. Primary analyses focused on the intended epidural analgesia group. Given anticipated analysis of at least five variables based on prior work [1], at least 50 women with intended epidural analgesia was targeted, assuming 10 events per variable are conventionally required in regression analyses. A power analysis for linear regression was conducted in StataSE 15 (StataCorp LP, 1985, College Station, TX) using 0.05 alpha, 15 tested predictors, and moderate effect size (f^2^ ≥ 15). With 55 epidural analgesia cases, there was 90% power to detect a significant association between EPDS and labor pain emotional burden.

#### Primary analysis: Univariable and multivariable analysis

Primary analyses focused on the intended epidural analgesia group. Simple linear regression was used to assess unadjusted relationships between six-week postpartum EPDS scores and demographic, psychological, obstetric, and pain variables. For multivariate analyses, covariates were selected based on a > 10% change in unadjusted and adjusted coefficients, as well as clinical judgement. Multicollinearity between variables was assessed using variance inflation factor > 10. Percentage variability in EPDS scores explained by demographic, psychological, obstetric, and pain variables was estimated using R^2^ coefficients in both the univariate and adjusted models.

Based on variable selection criteria described above, model adjustments included: baseline perceived social support (PSS), high NA (high depression and anxiety scores), last known cervical exam at epidural placement, labor duration, and African American race. PCS did not meet variable selection criteria and was not included in the multivariable models. A parsimonious decision was made to account for anxiety and depression scores using the combined construct of NA due to the high correlation of these variables in the clinical pain setting [21–26].

In an exploratory secondary analysis, the above univariable and multivariable analyses were repeated using the outcome of three-month postpartum EPDS scores.

#### Exploratory analysis: mediation modeling

Mediation analyses were performed to examine relationship between labor pain unpleasantness burden (predictor variable, X) and six-week EPDS scores (response variable, Y) in the labor analgesia cohort. A priori proposed potential mediators (mediator variable, M) in this relationship included labor pain management satisfaction and expectations, labor outcome, and postpartum PSSc. Similarly, potential mediators of the relationship between clinical history of anxiety/depression (predictor variable, X) and six-week EPDS scores (response variable, Y) were examined and focused on the cohort who planned and used epidural labor analgesia. A priori proposed potential mediators (mediator variable, M) included maximum labor pain intensity, maximum labor pain unpleasantness, labor pain unpleasantness burden, maximum pain at the six-week post-partum time point, and labor pain relief expectations.

Mediation modeling included mediator as a function of predictor (X➔M) and response as a function of both mediator and predictor (MX➔Y) [27]; X➔Y modeling was performed, followed by mediator statements (X➔M and MX➔Y). In other words, simple linear regression was used to assess relationships between predictor variable and proposed mediator and between proposed mediator and six-week EPDS scores. If both relationships were statistically significant (*P* < 0.05), multiple linear regression models including both predicator variable and proposed mediator were used to assess the presence of a mediation effect. Parameter estimates for direct and indirect effect of predictor X, 95% confidence intervals (CIs), and *P*-values were examined; significance of X➔M and MX➔Y models were defined as non-zero when both *P* < 0.05, using the Sobel-Goodman test for mediation followed by bootstrap resampling for calculating CIs in SAS; the parameter estimates (β) and overall C statistic with 95% CI for the mediation models were reported.

#### Exploratory analysis: moderation modeling

Several variables were tested for moderating effect on relationship between labor pain unpleasantness burden and six-week EPDS scores. Cross product interaction terms were created between pain unpleasantness burden and potential moderators, and interaction term significance was assessed using linear regression with six-week EPDS scores as the outcome [28]. No-epidural group data was used and included moderation modeling to assess the influence of analgesic preference. Additionally, in the epidural labor analgesia cohort, high NA, African American race, pain catastrophizing, and PSS were examined as potential moderators of this relationship. Parameter estimates (β) and 95% CIs were reported. Cohort characteristics were compared between epidural groups using Chi-Square statistic, Wilcoxon rank sum, and Fisher exact and unpaired student t-tests where appropriate. All tests were two-sided and *P* < 0.05 was considered statistically significant. Analyses were performed using StataSE 15.0 SE (StataCorp LP, 1985, College Station, TX) and SAS version 9.4 statistical software (SAS Institute Inc., Cary, NC).

## Results

**Fig. 1:**
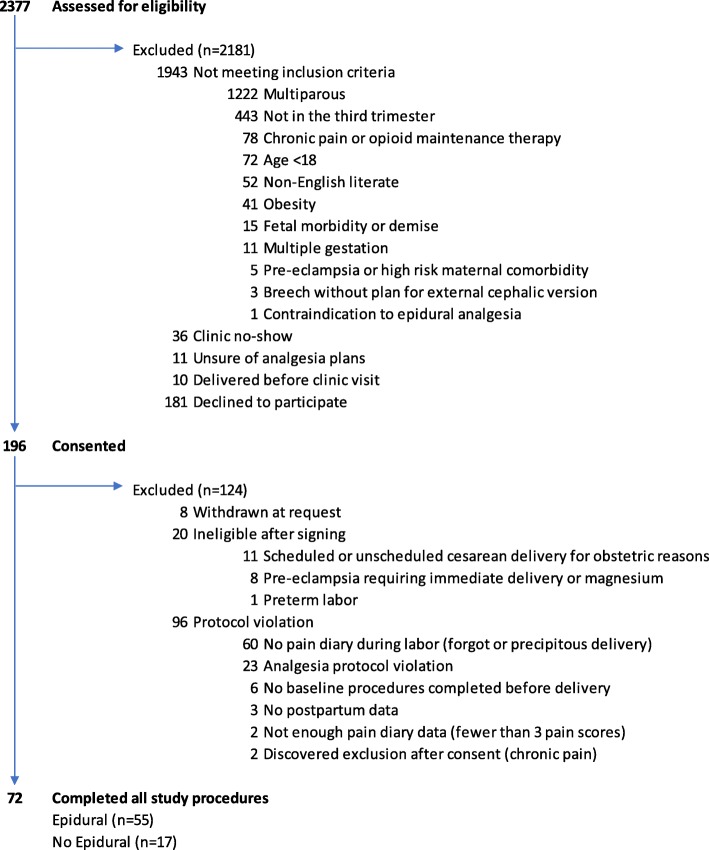
Study flow diagram

72 participants were in the final cohort (Fig. 1); of these, 55 received epidural labor analgesia and 17 received no epidural labor analgesia. All women in the final cohort received their planned analgesia method.

Measured baseline, labor, and postpartum characteristics did not differ between women receiving and avoiding epidural labor analgesia (Table 1). Pain management satisfaction scores were no different between epidural (median = 1.9, IQR 1.4–2.1) and no-epidural (median = 1.9, IQR 1.5–2.1) groups (z = 0.33, *P* = 0.74) (score of 2 = “satisfied”). Similarly, labor pain management expectation scores were no different between epidural (median = 2.2, IQR 1.5–2.5) and no-epidural (media*n* = 3, IQR 2–3) groups (z = 1.9, *P* = 0.06) (score of 2=“somewhat exceeds my expectations”, 3=“meets my expectations”). PCS, ER-89, PSS, and PSSc scores did not differ between groups. EPDS score ranges were in the following categories as follows: 9 or less, low depression concerns (*n* = 65, 90.3%); 10–12, modest concern (*n* = 3, 4.2%); 13–18, moderate concern (*n* = 3, 4.2%); 19 and above, likely to have depression, concern and worry about suicide risk (*n* = 1, 1.4%).

**Table 1:** Demographic, psychosocial, pain, and obstetric characteristics comparing cohort of women receiving and avoiding epidural labor analgesia

	No Epidural (***n*** = 17)	Epidural (***n*** = 55)	***P***-value
**Demographic Characteristics**			
*Age (years)*	30.6 (4.3)	30.1 (4.8)	0.69
*Body mass index (kg/m*^*2*^*)*	29.7 (6.0)	32.2 (5.3)	0.20
*Gravidity*			
1	14 [82.4]	43 [78.2]	0.99
2	2 [11.8]	9 [16.4]	
3	1 [5.9]	2 [3.6]	
4	0 [0]	1 [1.8]	
*Estimated gestational age (weeks)*	40 {0,40,40}	40{1,39,40}	0.23
*Race*			
American Indian	0 [0]	2 [3.6]	0.99
Asian	0 [0]	4 [7.3]	0.57
African American	2 [11.8]	9 [16.4]	0.99
White	16 [94.1]	44 [80]	0.27
Other	0 [0]	1 [1.8]	0.99
*Ethnicity*			
Hispanic/Latino	0 [0]	4 [7.4]	0.57
Non-Hispanic/Latino	17 [100]	50 [92.6]	
**Obstetric Variables**			
*Mode of delivery*			
Normal spontaneous vaginal delivery	15 [88.2]	41 [74.5]	0.95
Assisted vaginal - forceps	0 [0]	0 [0]	
Assisted vaginal - vacuum	0 [0]	2 [3.6]	
Cesarean – non-reassuring fetus	1 [5.9]	3 [5.5]	
Cesarean - arrest of dilation/descent	1 [5.9]	7 [12.7]	
Cesarean - other	0 [0]	2 [3.6]	
*Perineal lacerations*			
None	5 [29.4]	16 [29.63]	0.99
First Degree	3 [17.6]	8 [14.8]	
Second Degree	9 [52.9]	27 [50]	
Third Degree	0 [0]	2 [3.7]	
Fourth Degree	0 [0]	1 [1.8]	
*Duration of labor (hours)*	15.7 (6.3)	18.6 (8.5)	0.19
**Psychological Variables**			
History of Anxiety or Depression	8 [50.00]	17 [30.9]	0.16
History of Mental Illness	1 [6.25]	6 [10.9]	0.99
EPDS Baseline	4 {6,1,7}	3 {6,1,7}	0.62
EPDS 6 weeks	3 {5,2,7}	3 {5,1,6}	0.76
EPDS 3 months	4.1 (3.6)	4.1 (3.8)	0.93
Anxiety State (STAI/state)	33.1 (8.1)	34.1 (10.4)	0.72
Anxiety Trait (STAI/trait)	33.2 (7.6))	33.6 (10.1)	0.86
Resiliency (ER-89)	45.5 (5.9)	42.8 (6.3)	0.13
Pain Catastrophizing Scale (PCS)	10.5 (8.8)	10.5 (8.8)	0.99
Perceived Social Support (PSS)	6.5 {0.8,5.9,6.7}	6.5 {1.3,5.8,7}	0.68
Perceived Stress Scale (PSSc)	7 {0,7,7}	7 {0.8,6.3,7}	0.09
**Employment status**			
Employed outside home, full-time	13 [76.5]	37 [67.2]	0.80
Employed outside home, part-time	1 [5.9]	8 [14.5]	
Homemaker	2 [11.8]	3 [5.5]	
Retired	0 [0]	0 [0]	
Unemployed	1 [5.9]	5 [9.1]	
Other	0 [0]	2 [3.6]	
**Marital status**			
Single	2 [11.8]	15 [27.3]	0.40
Married	15 [88.2]	39 [70.9]	
Widowed	0 [0]	0 [0]	
Divorced	0 [0]	1 [1.8]	
**Labor Pain, Satisfaction, & Expectations**			
Baseline labor pain score (mm)	7.1 (4.6)	7.2 (7.1)	0.94
Post-epidural analgesia average pain score (mm)	–	9.1 (5.6)	–
Labor pain intensity max score (mm)	84.1 (14.4)	75.9 (23.0)	0.17
Labor pain unpleasantness max score (mm)	86.3 (12.0)	77.9 (22.3)	0.14
Labor pain intensity burden (AUC)	452.4 (316.2)	410.3 (240.3)	0.56
Labor pain unpleasantness burden (AUC)	450.9 (675.4)	428.6 (276.1)	0.78
Labor pain management satisfaction (score)	1.9 (0.6)	1.8 (0.4)	0.52
Labor pain management expectation (score)	2.4 (0.9)	2.1 (0.7)	0.12

Data are reported as mean (standard deviation), frequency [percent], or median {interquartile range, 25th percentile, 75th percentile}. *EPDS* Edinburgh Postnatal Depression Scale, *STAI* State Trait Anxiety Inventory, *ER-89* Ego Resiliency Scale, *PCS* Pain Catastrophizing Scale, *PSS* Perceived Social Support Scale, *PSSc* Perceived Stress Scale, *AUC* Area under curve

Univariable analysis assessed relationship between demographic, obstetric, and prenatal psychiatric and social variables and six-week postpartum EPDS score among women planning and receiving labor epidural analgesia (Table 2). Relationships significantly predicting six-week EPDS score were history of anxiety or depression (R^2^ = 0.20, 95% CI 1.98–4.55, *P* = 0.0007), baseline depression score (R^2^ = 0.24, 95% CI 0.27–0.75, *P* = 0.0002), anxiety state score (R^2^ = 0.20, 95% CI 0.09–0.29, *P* = 0.0005), trait score (R^2^ = 0.19, 95% CI 0.08–0.29, *P* = 0.001), high NA (R^2^ = 0.09, 95% CI 0.42–6.32, *P* = 0.03), low NA (R^2^ = 0.11, 95% CI − 5.16 to − 0.59, *P* = 0.01), PSS (R^2^ = 0.17, 95% CI − 2.77 to − 0.67, *P* = 0.002), and African American race (R^2^ = 0.07, 95% CI 0.10–6.30, *P* = 0.04). Delivery mode and breastfeeding outcomes were not associated with 6 weeks postpartum EPDS scores. Other measured demographic and obstetric variables did not associate with six-week PPD scores.

**Table 2:** Univariable analysis assessing relationship between prenatal, labor and delivery variables and EPDS score at 6 weeks postpartum, among women planning and receiving labor epidural analgesia (*n* = 55)

Variable	R^2^	Confidence Interval	*P*-value
**Demographics**			
Age (years)	0.01	−0.19 to 0.31	0.62
American Indian	0.001	−6.94 to 5.81	0.86
Asian	0.002	−3.83 to 5.35	0.74
African American	0.07	0.10 to 6.30	0.04*
White	0.07	−5.82 to −0.08	0.05
Other	0.01	−11.49 to 6.31	0.56
Hispanic ethnicity	0.01	−6.32 to 2.88	0.46
**Obstetric Variables**			
Estimated gestational age (weeks)	0.02	−1.46 to 0.51	0.34
Gravidity	0.06	−0.21 to 3.51	0.08
Duration of labor (hours)	0.02	−0.06 to 0.22	0.26
Cervical exam at time of epidural request (cm)	0.01	−0.89 to 0.38	0.43
Mode of Delivery	NS	NS	NS
Perineal lacerations	NS	NS	NS
*Breastfeeding*			
Breastfeeding Postpartum 1–2 Days	0.02	−2.54 to 7.98	0.30
Breastfeeding Postpartum 6 Weeks	0.02	−5.34 to 1.89	0.34
**Prenatal Psychiatric Variables**			
History of Anxiety or Depression	0.20	1.98 to 4.55	0.0007*
History of Mental Illness	0.09	0.61 to 7.90	0.02*
Baseline Depression Score	0.24	0.27 to 0.79	0.0002*
Anxiety-State	0.20	0.09 to 0.29	0.0005*
Anxiety-Trait	0.19	0.08 to 0.29	0.001*
High Negative Affect	0.09	0.42 to 6.32	0.03*
Moderate Negative Affect	0.01	−1.69 to 3.89	0.43
Low Negative Affect	0.11	−5.16 to −0.59	0.01*
Pain Catastrophizing	0.03	−0.05 to 0.22	0.24
Resiliency	0.03	−0.30 to 0.08	0.24
Perceived Stress Scale	0.08	−0.02 to 1.0	0.06
**Prenatal Social Variables**			
Perceived Social Support	0.17	−2.77 to −0.67	0.002*
Employment Status	NS	NS	NS
Marital Status	NS	NS	NS

*P < 0.05

*NS* Not significant

Labor pain unpleasantness burden predicted six-week EPDS score (*P* = 0.0008). Several other perinatal pain variables were identified on univariable analysis as predictive of six-week EPDS score (Table 3). These included prenatal pain variables (least pain in the last week, *P* = 0.02, and pain returns 2 hours after taking medication, *P* = 0.0002); labor pain variables (labor pain intensity burden (*P* = 0.005), and postpartum pain variables (six-week measurements for “pain today (*P*=0.0013),” “pain at its worst in the past 24 hours (*P*=0.0007),” “pain on average (*P*=0.0028),” and “pain right now (*P*=0.0006)”).

**Table 3:** Univariable analysis assessing relationship between pain variables and EPDS score at 6 weeks among women planning and receiving labor epidural analgesia (*n* = 55)

Pain Variable	R^2^	Confidence Interval	*P*-value
***Prenatal Pain***			
Severity & Impact of Pain on Function			
Pain at its ‘least’ in the last week	0.22	0.14 to 1.69	0.02*
Pain returns 2 h after taking medication	0.56	8.53 to 22.58	0.0002*
***Labor Pain***			
Labor Pain Intensity Burden	0.14	0.00 to 0.01	0.005*
Labor Pain Unpleasantness Burden	0.19	0.00 to 0.01	0.0008*
***Postpartum Pain***			
Pain at 6 weeks - pain today?	0.19	1.90 to 7.36	0.0013*
Pain at 6 weeks - pain at worst past 24 h?	0.21	0.43 to 1.50	0.0007*
Pain at 6 weeks - pain on average?	0.17	0.33 to 1.48	0.0028*
Pain at 6 weeks - pain right now?	0.22	0.49 to 1.69	0.0006*
Pain at 6 weeks – Interference with:			
General activity	0.28	2.29 to 4.64	< 0.0001*
Mood	0.39	0.91 to 1.95	< 0.0001*
Walking ability	0.27	0.56 to 1.57	< 0.0001*
Normal work	0.27	0.66 to 1.91	< 0.0001*
Relations with other people	0.54	1.35 to 2.34	< 0.0001*
Sleep	0.25	0.54 to 1.57	0.0002*
Enjoyment of life	0.47	0.93 to 1.76	< 0.0001*

*P < 0.05

**---***Other pain variables that were measured at these time points that were not significant are available on request*

**Fig. 2:**
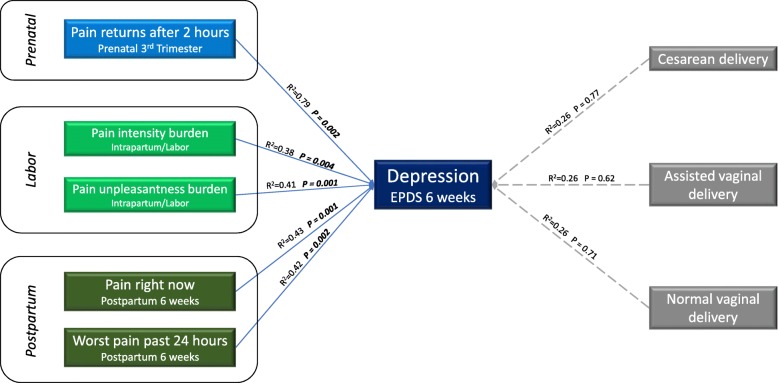
Multivariable analysis assessing the relationship between prenatal, labor, and postpartum pain factors and postpartum depression scores at 6 weeks among women planning and receiving epidural analgesia. Pain at each perinatal time point (prenatal, labor, and postpartum) was independently associated with depression scores at 6 weeks. Mode of delivery was not a significant predictor for depression scores at 6 weeks. Models were adjusted for perceived social support, baseline high negative affect (high depression and high anxiety scores), last known cervical exam at the time of epidural analgesia initiation, duration of labor, and African American race

After adjusting for covariates (baseline PSS, high NA, last known cervical exam at time of epidural placement, labor duration, African American race), pain at each perinatal time point (prenatal, labor, postpartum) was independently associated with six-week depression scores (Fig. 2).

**Fig. 3:**
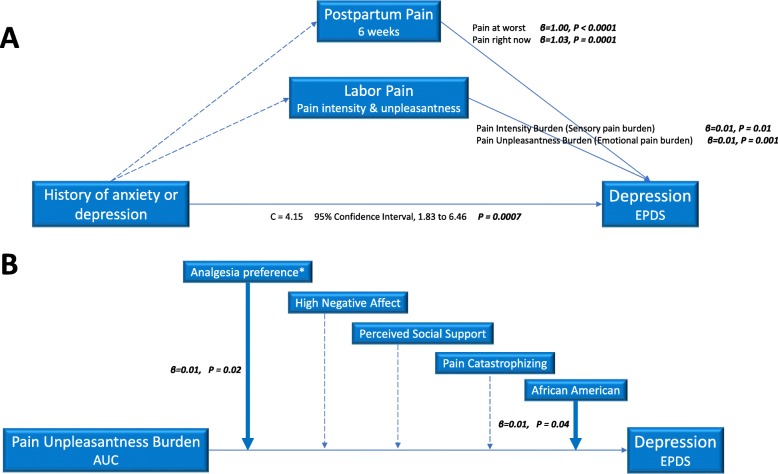
Mediation and moderation effects between variables of interest. (A) Mediation effects between a history of anxiety or depression and postpartum depression scores. The relationship between prenatal anxiety or depression and postpartum depression scores was not mediated by labor pain and postpartum pain. Labor pain and postpartum pain remained independent predictors of six-week postpartum depression scores (solid lines). Relationships that are not significant are indicated by dotted lines. (B) Moderation effects between the emotional burden of labor pain and postpartum depression scores. The strength of the relationship between the emotional burden of labor pain and postpartum depression increases for women choosing and receiving epidural analgesia and for women who are African American (solid lines). The strength of the relationship did not change for negative affect, perceived social support, or pain catastrophizing (dotted lines). AUC, area under the curve; EPDS, Edinburgh Postnatal Depression Scale

Mediation and moderation effects were assessed (Fig. 3). Relationship between labor pain unpleasantness burden and six-week PPD scores was significant (C statistic = 0.005, 95% CI, 0.001–0.01, *P* = 0.01); this relationship was not mediated by labor pain management satisfaction or expectations, postpartum perceived stress, nor delivery mode (Supplemental Table 1a). Relationship between prenatal clinical anxiety or depression and six-week PPD scores was significant (C statistic = 4.15, 95% CI, 1.83–6.46, *P* = 0.0007) and not mediated by labor or postpartum pain (Supplemental Table 1b).

For moderation effects, strength of the relationship between labor pain emotional burden and six-week PPD scores increased for women choosing and receiving epidural analgesia (estimate = 0.01, 95% CI 0.001–0.02, *P* = 0.02). Among women planning and receiving labor epidural analgesia, strength of the relationship between labor pain emotional burden and PPD scores increased for African Americans (estimate = 0.01, 95% CI 0.00–0.02, *P* = 0.04). Relationship strength did not change for prenatal NA, PSS, or pain catastrophizing (Supplemental Table 2).

For exploratory analysis using the outcome of three-month depression scores in the labor epidural analgesia cohort, findings showed a similar and statistically significant relationship between labor and sub-acute (six-week) postpartum pain and three-month depression scores. After adjusting for PSS, high prenatal NA, last known cervical dilation at labor epidural analgesia initiation, labor duration, and African American race, the following factors were significant independent predictors of three-month depression scores (Supplemental Table 3): labor pain intensity burden, labor pain unpleasantness burden, six-week “pain right now” scores (BPI-S #6), six-week “pain at worst in the past 24 hours” scores (BPI-S #3), sulcus lacerations, and several six-week postpartum pain interference assessments (BPI-S #9) (general activity, mood, walking ability, normal work, relationships, sleep, enjoyment of life). Three-month postpartum pain outcomes (walking ability and 24-h worst pain) as well as delivery mode (cesarean, normal spontaneous vaginal, assisted vaginal) did not significantly predict three-month postpartum depression scores (Supplemental Table 3).

## Discussion

Key findings from this prospective cohort study are that, in a carefully selected cohort of women planning and receiving epidural labor analgesia: 1) perinatal pain at all time points – prenatal, labor, and postpartum – independently predicts six-week PPD scores; 2) strength of the relationship between labor pain and PPD scores increases for African American race and labor analgesia preference; and 3) labor and acute postpartum pain predict six-week and three-month postpartum PPD scores, but pain approaching the “chronic” three-month period does not predict three-month PPD scores. These relationships do not appear driven by labor pain management satisfaction or expectations.

Depression in the postpartum period typically starts during the first 6 weeks after delivery but can occur any time during the first 12 months after delivery. 50–80% of women experience transient “baby blues,” 0.1 to 0.2% of women experience postpartum psychosis usually within the first 4 weeks following delivery [19, 29, 30]. PPD impacts infant and childhood development, including delayed cognitive and psychological development, impaired vocalization, delayed motor skills, increased healthcare resource use and the marriage and partnerships. Symptoms range from mild dysphoria to suicidal ideation, to psychotic depression. Babies with mothers with untreated perinatal depression show neurobehavioral changes and have elevated stress hormones. Epigenetic changes and neurobiological models of emotion-behavior regulation are implicated during the first 1000 days of life; attention regulation capacities that call on brainstem and limbic system networks are affected.

The current study builds knowledge about relationships between labor and acute postpartum pain and depression. Eisenach et al. [3] linked severity of acute postpartum pain, but not delivery mode, to eight-week postpartum PPD scores; every point increase in acute pain after delivery was associated with an 8.3% increase in eight-week depressive symptoms. The current study confirms these findings; we did not find a relationship between delivery mode (i.e. degree of tissue injury) and PPD scores. Our findings add to these former findings. Although we detected a relationship between labor and acute (six-week) postpartum pain variables and both six-week and three-month postpartum EPDS scores, three-month pain variables did not correlate with three-month EPDS scores. These findings seem to suggest that acute and sub-acute pain may play a vital role in downstream vulnerability to mood disorders in a chronic (three-month) period.

Our data may indicate that labor and acute postpartum pain are important constructs in a paradigm describing acute events (pain) as a trigger for transition to long-term risk (depression). They also indicate that underlying biological explanations for these relationships between pain and depression aren’t necessarily rooted in degree of tissue injury. We reproduced former findings [3] that failed to show a significant effect of delivery mode on PPD risk. Rather, our findings suggest that psychological aspects of the pain *experience*, as opposed to clinical factors associated with higher pain levels, are important variables in evaluating PPD risk. Screening for PPD is important, and in addition to the EPDS, in the primary care setting a simple tool is often used with 97% sensitivity, 67% specificity by two questions: 1) During the past month, have you been bothered by little interest or pleasure in doing things? 2) During the past months, have you often been feeling down, depressed, or hopeless [31].

Results of our moderation-mediation analyses are informative. Although this is the first study demonstrating a moderation effect of race on relationship between labor pain and depression to our knowledge, the findings may not necessarily be surprising given other known areas of health disparities. Racial and ethnic pain and pain treatment disparities are well-described, with African American and Hispanic/Latino minorities known to bear higher acute, perioperative, and chronic pain burdens [32–34]. Potential factors explaining these observations include patient-level factors (differences in pain perception/nociceptive processing among races; genetic factors; pain attitudes and beliefs) [33, 35–38]. Our results also showed that labor epidural analgesia preference strengthened the relationship between labor pain emotional valence (labor pain unpleasantness burden) and PPD scores. These findings may be explained by factors like fear of childbirth that potentially influence labor epidural analgesia choice in some cultures [39]. A systematic review and meta-analysis comparing effects of continuous intrapartum support to usual care showed a reduction in analgesia utilization and poor outcomes such as negative feelings about childbirth experiences [40]. However, whether these interventions have definitive preventative effects for PPD is unknown [40]; which specific vulnerable population sub-groups could be harmed by lack of provision of functional labor analgesia when desired (such as in low-resource settings) are even less clear.

The current results did not show a mediation effect for labor and postpartum pain on the relationship between baseline anxiety or depression and PPD scores. Also, for the relationship between labor pain unpleasantness burden and PPD scores, there was no mediation effect for pain management expectations/satisfaction, perceived stress, and delivery mode. These findings are important because they provide further evidence supporting an independent relationship between labor/acute postpartum pain variables and PPD risk. Among women planning and receiving labor analgesia, both sensory and affective/emotional aspects of labor pain seem to be important to postpartum recovery. The basis for this relationship and diagnostic/treatment ramifications warrants additional investigation.

Our findings complement those from Orbach-Zinger and colleagues [8], who found that unmatched intention effects with respect to *use of* labor analgesia are significantly associated with six-week depression risk. The current study focused on the pain experience of women planning and receiving their intended labor analgesia approach. In this carefully selected cohort of women planning and receiving labor epidural analgesia, perinatal pain variables were independently associated with depression risk. The present cohort had prospective evaluations of labor pain management expectations and satisfaction. These findings indicate that even when labor pain management satisfaction and expectations are largely met or exceeded, labor pain is still an important factor for PPD symptoms. Mediation analysis results also indicate that expectations and satisfaction were not explanatory factors in the relationship between labor pain unpleasantness burden (emotional burden of pain) and six-week PPD scores.

Prenatal PCS scores in this cohort did not meet variable selection criteria for our multivariable modeling. Similarly, Carvalho et al. [41] did not find that PCS met multivariable regression model selection criteria for labor pain. Also, in a study of preoperative predictive tests in cesarean delivery [42], PCS was not significantly correlative to post-cesarean delivery pain or opioid requirements. These findings may be surprising, because other studies have found correlation between higher PCS scores and higher labor pain intensity and postpartum pain scores [17, 43, 44], and pain catastrophizing and neuroticism are known to be strongly linked to physical disability and poor quality of life outcomes in chronic pain populations [45]. An exploratory analysis where PCS was included in the multivariable modeling did not meaningfully change the present study’s findings (results not shown). Sample size may explain failure of PCS to meet variable selection criteria in our cohort.

Study limitations included potential sampling bias; some subjects would not participate due to unwillingness to log hourly pain scores during labor. Omission of this group could impact findings in that these patients may represent: 1) people so averse to pain that they desire avoiding all conscious recognition of it; or 2) people whose resilience/attitudes toward labor pain that they embrace its necessity and desire developing robust coping strategies, possibly including avoidance of explicit pain cognition. Hourly pain assessments could directly conflict with the latter approach. This omission highlights the challenges of conducting pain and analgesia research on this population and underscores the profound need to develop practical methodologies to assess such limitations in future work. Because we focused on labor pain for this study, we excluded women undergoing cesarean delivery without experiencing labor. By omitting these patients, potential unmet birth experience expectations may differentially impact PPD risk, as suggested by Orbach-Zinger et al. [8]. However, we reason that the validity of our findings is not significantly impacted by this omission, as we sought to answer questions specifically about labor pain, its management, and expectations without focusing on delivery mode. Furthermore, and consistent with our own findings in the present study, other investigations have noted that delivery mode did not predict PPD [3].

## Conclusions

In summary, we describe findings supporting a model in which acute pain (labor and postpartum) variables predict PPD symptoms at short- and long-term intervals (six-week and three-month postpartum EPDS scores). Among women electing and receiving labor epidural analgesia, the entire perinatal pain experience is linked to PPD symptoms; the relationship’s strength is influenced by race and labor analgesia preference factors. These relationships are not mediated by labor pain management satisfaction or expectations. Psychological and cognitive-affective aspects of perinatal pain, analgesia preference, and their influence on depression risk should be addressed in subsequent investigations. Integrating primary care with mental health services have been shown to improve overall medical care and reduces costs. This study sheds new knowledge on pain as a potential variable of independent predictive interest in PPD symptoms.

## Supplementary information

**Additional file 1: Supplemental tables**. These tables give detailed results of the mediation and moderation analyses as well as the multivariate regression analyses.

**Additional file 2: Supplemental flow diagram**. This diagram shows the specific variables assessed at each specific timepoint.

## Data Availability

The datasets used and analyzed during the current study are available from the corresponding author on reasonable request.

## Abbreviations

PPD: Postpartum depression
EPDS: Edinburgh postnatal depression scale
PCS: Pain catastrophizing scale
BPI: Brief pain inventory (short, S and long, L)
PSS: Perceived social support
NA: Negative affect
PSSc: Perceived stress scale
ER-89: Ego-resiliency scale
AUC: Area under curve
PTSS: Pain treatment satisfactions cale
STAI: State trait anxiety inventory
STROBE: Strengthening the reporting of observational studies in epidemiology
