# Supplementary material for: Obstetric pain correlates with postpartum depression symptoms: a pilot prospective observational study
Source: BMC Pregnancy Childbirth. 2020 Apr 22;20:240. doi: 10.1186/s12884-020-02943-7 (PMC7178606; doi:10.1186/s12884-020-02943-7)
Supplement: Supplementary file 1 — Additional file 1: Supplemental tables. These tables give detailed results of the mediation and moderation analyses as well as the multivariate regression analyses. [file 12884_2020_2943_MOESM1_ESM.docx]

| **Supplemental Table 1a. Mediation effects for relationship between labor pain unpleasantness burden and six-week EPDS scores among women planning and receiving epidural analgesia** | | | | |
| --- | --- | --- | --- | --- |
|  | Parameter Estimate | 95% CI | P-value | Notes |
| Pain management satisfaction during labor |  |  |  |  |
| Labor pain unpleasantness burden-> EPDS 6wk | 0.005 | 0.001 to 0.01 | 0.01 |  |
| Labor pain unpleasantness burden -> Pain manage satisfaction | 0.001 | 0.0002 to 0.001 | 0.004 |  |
| Pain manage satisfaction -> EPDS 6wk | 1.13 | -1.12 to 3.37 | 0.32 | Likely no mediation because this relationship is not significant |
|  |  |  |  |  |
| Complete model: |  | Standard Error |  |  |
| Labor pain unpleasantness burden | 0.005 | 0.002 | 0.008 |  |
| Pain manage satisfaction | 1.13 | 1.13 | 0.32 | Relationship not significant, no evidence of mediation |
|  |  |  |  |  |
| Sobel Test | test stat: 0.0006 |  | 0.34 | NOT SIGNIFICANT MEDIATION USING SOBEL TEST |
| Pain management expectations during labor |  |  |  |  |
| Labor pain unpleasantness burden-> EPDS 6wk | 0.004 | 0.001 to 0.01 | 0.017 |  |
| Labor pain unpleasantness burden -> Pain manage expectations | 0.001 | 0.0003 to 0.001 | 0.003 |  |
| Pain manage expectations -> EPDS 6wk | -0.35 | -1.9 to 1.2 | 0.65 | Likely no mediation because this relationship is not significant |
|  |  |  |  |  |
| Complete model: |  | Standard Error |  |  |
| Labor pain unpleasantness burden | 0.004 | 0.002 | 0.01 |  |
| Pain manage expectations | -0.35 | 0.78 | 0.65 | Relationship not significant, no evidence of mediation |
|  |  |  |  |  |
| Sobel Test | test stat: -0.0003 |  | 0.65 | NOT SIGNIFICANT MEDIATION USING SOBEL TEST |
| Perceived stress scale (postpartum day 1-2) |  |  |  |  |
| Labor pain unpleasantness burden-> EPDS 6wk | 0.005 | 0.001 to 0.009 | 0.006 |  |
| Labor pain unpleasantness burden -> Perceived stress scale | -0.054 | -0.15 to 0.042 | 0.26 | Likely no mediation because this relationship is not significant |
| Perceived stress scale -> EPDS 6wk | -0.0057 | -0.015 to 0.0038 | 0.24 | Likely no mediation because this relationship is not significant |
|  |  |  |  |  |
| Complete model: |  | Standard Error |  |  |
| Labor pain unpleasantness burden | 0.005 | 0.0018 | 0.005 |  |
| Perceived stress scale | -0.0057 | 0.0047 | 0.23 |  |
|  |  |  |  |  |
| Sobel Test | test stat: 0.0003 |  | 0.41 | NOT SIGNIFICANT MEDIATION USING SOBEL TEST |
| Mode of delivery |  |  |  |  |
| Labor pain unpleasantness burden-> EPDS 6wk | 0.005 | 0.001 to 0.008 | 0.01 |  |
| Labor pain unpleasantness burden -> Mode of delivery | 0.0007 | -0.0007 to 0.002 | 0.32 | Likely no mediation because this relationship is not significant |
| Mode of delivery -> EPDS 6wk | -0.17 | -0.79 to 0.45 | 0.59 | Likely no mediation because this relationship is not significant |
|  |  |  |  |  |
| Complete model: |  | Standard Error |  |  |
| Labor pain unpleasantness burden | 0.005 | 0.002 | 0.01 |  |
| Mode of delivery | -0.17 | 0.001 | 0.32 |  |
|  |  |  |  |  |
| Sobel Test | test stat: -0.0001 |  | 0.64 | NOT SIGNIFICANT MEDIATION USING SOBEL TEST |

*EPDS, Edinburgh postnatal depression scale. BPI, brief pain inventory. AUC, area under curve.*

**Supplemental Table 1b. Mediation effects for relationship between history of anxiety/depression and six-week EPDS scores among women planning and receiving epidural analgesia**

|  | Parameter Estimate | 95% CI | P-value | Notes |
| --- | --- | --- | --- | --- |
| Pain Intensity Max |  |  |  |  |
| History of Anxiety/Depression -> EPDS 6wk | 4.15 | 1.83 to 6.46 | 0.0007 |  |
| History of Anxiety/Depression -> Pain Intensity Max | 4.27 | -9.26 to 17.80 | 0.53 | Likely no mediation because this relationship is not significant |
| Pain Intensity Max -> EPDS 6wk | 0.03 | -0.02 to 0.09 | 0.19 | Likely no mediation because this relationship is not significant |
|  |  |  |  |  |
| Complete model: |  |  |  |  |
| History of Anxiety/Depression | 4.03 | 1.71 to 6.35 | 0.001 |  |
| Pain Intensity Max | 0.03 | -0.02 to 0.07 | 0.25 | Relationship not significant, no evidence of mediation |
|  |  |  |  |  |
| Sobel Test | test stat: 0.57 |  | 0.57 | NOT SIGNIFICANT MEDIATION USING SOBEL TEST |
| Pain Unpleasantness Max |  |  |  |  |
| History of Anxiety/Depression -> EPDS 6wk | 4.15 | 1.83 to 6.46 | 0.0007 |  |
| History of Anxiety/Depression -> Pain Unpleasantness Max | 5.38 | -7.68 to 18.44 | 0.41 | Likely no mediation because this relationship is not significant |
| Pain Unpleasantness Max -> EPDS 6wk | 0.05 | 0.00 to 0.11 | 0.04 |  |
|  |  |  |  |  |
| Complete model: |  |  |  |  |
| History of Anxiety/Depression | 3.91 | 1.63 to 6.19 | 0.001 |  |
| Pain Unpleasantness Max | 0.04 | 0.00 to 0.09 | 0.07 | Relationship not significant, no evidence of mediation |
|  |  |  |  |  |
| Sobel Test | test stat: 0.77 |  | 0.44 | NOT SIGNIFICANT MEDIATION USING SOBEL TEST |
| AUC Unpleasantness |  |  |  |  |
| History of Anxiety/Depression -> EPDS 6wk | 4.15 | 1.83 to 6.46 | 0.0007 |  |
| History of Anxiety/Depression -> AUC Unpleasantness | 1.69 | -24.67 to 289.66 | 0.1 | Likely no mediation because this relationship is not significant |
| AUC Unpleasantness -> EPDS 6wk | 0.007 | 0.00 to 0.01 | 0.0008 |  |
|  |  |  |  |  |
| Complete model: |  |  |  |  |
| History of Anxiety/Depression | 3.39 | 1.18 to 5.60 | 0.003 |  |
| AUC Unpleasantness | 0.006 | 0.00 to 0.01 | 0.004 |  |
|  |  |  |  |  |
| Sobel Test | test stat: 1.53 |  | 0.13 | NOT SIGNIFICANT MEDIATION USING SOBEL TEST |
| AUC Intensity |  |  |  |  |
| History of Anxiety/Depression -> EPDS 6wk | 4.15 | 1.83 to 6.46 | 0.0007 |  |
| History of Anxiety/Depression -> AUC Intensity | 93.2 | -46.42 to 232.87 | 0.19 | Likely no mediation because this relationship is not significant |
| AUC Intensity -> EPDS 6wk | 0.006 | 0.0013 to 0.0099 | 0.013 |  |
|  |  |  |  |  |
| Complete model: |  |  |  |  |
| History of Anxiety/Depression | 3.39 | 1.18 to 5.60 | 0.003 |  |
| AUC Intensity | 0.006 | 0.00 to 0.01 | 0.004 |  |
|  |  |  |  |  |
| Sobel Test | test stat: 1.53 |  | 0.13 | NOT SIGNIFICANT MEDIATION USING SOBEL TEST |
| BPI Short # 6 @ 6wks (pain right now) |  |  |  |  |
| History of Anxiety/Depression -> EPDS 6wk | 4.15 | 1.83 to 6.46 | 0.0007 |  |
| History of Anxiety/Depression -> BPI Short 6 | 0.26 | -0.89 to 1.40 | 0.65 | Likely no mediation because this relationship is not significant |
| BPI Short 6 -> EPDS 6wk | 1.09 | 0.49 to 1.69 | 0.0006 |  |
|  |  |  |  |  |
| Complete model: |  |  |  |  |
| History of Anxiety/Depression | 3.76 | 1.61 to 5.92 | 0.001 |  |
| BPI Short 6 | 1.03 | 0.49 to 1.57 | 0.0004 |  |
|  |  |  |  |  |
| Sobel Test | test stat: 0.45 |  | 0.65 | NOT SIGNIFICANT MEDIATION USING SOBEL TEST |
| BPI Short # 3 @ 6wks (pain at worst) |  |  |  |  |
| History of Anxiety/Depression -> EPDS 6wk | 4.15 | 1.83 to 6.46 | 0.0007 |  |
| History of Anxiety/Depression -> BPI Short 3 | -0.19 | -1.47 to 1.09 | 0.77 | Likely no mediation because this relationship is not significant |
| BPI Short 3 -> EPDS 6wk | 0.96 | 0.43 to 1.50 | 0.0007 |  |
|  |  |  |  |  |
| Complete model: |  |  |  |  |
| History of Anxiety/Depression | 4.22 | 2.13 to 6.30 | 0.0002 |  |
| BPI Short 3 | 1 | 0.54 to 1.47 | <.0001 |  |
|  |  |  |  |  |
| Sobel Test | test stat: -0.30 |  | 0.77 | NOT SIGNIFICANT MEDIATION USING SOBEL TEST |
| Relief Expectation |  |  |  |  |
| History of Anxiety/Depression -> EPDS 6wk | 4.15 | 1.83 to 6.46 | 0.0007 |  |
| History of Anxiety/Depression -> Relief Exp | 0.15 | -0.24 to 0.53 | 0.45 | Likely no mediation because this relationship is not significant |
| Relief Exp -> EPDS 6wk | 1.39 | -0.42 to 3.19 | 0.13 | Likely no mediation because this relationship is not significant |
|  |  |  |  |  |
| Complete model: |  |  |  |  |
| History of Anxiety/Depression | 3.99 | 1.68 to 6.30 | 0.001 |  |
| Relief Exp | 1.09 | -0.56 to 2.74 | 0.19 |  |
|  |  |  |  |  |
| Sobel Test | test stat: 0.68 |  | 0.49 | NOT SIGNIFICANT MEDIATION USING SOBEL TEST |
|  |  |  |  |  |

**Supplemental Table 2. Moderation effects between labor pain unpleasantness burden and six-week EPDS score, for analgesia preference (overall cohort) and NA, PSS, PCS, and African American Race (epidural cohort).**

|  | Parameter Estimate | 95% CI | P value | Notes |
| --- | --- | --- | --- | --- |
| Epidural*Pain Unpleasantness AUC | |  |  |  |
| Epidural | -3.85 | -7.78 to 0.09 | 0.06 |  |
| Pain Unpleasantness AUC | -0.002 | -0.008 to 0.004 | 0.58 |  |
| Epidural*AUC | 0.009 | 0.001 to 0.02 | 0.02 | Significant interaction: wanting/receiving labor epidural analgesia increases the effect of labor pain unpleasantness burden on six-week postpartum EPDS score |
|  |  |  |  |  |
| High Negative Affect in the Epidural Group | | |  |  |
| High Neg Affect | 1.98 | -3.36 to 7.33 | 0.46 |  |
| Pain Unpleasantness AUC | 0.006 | 0.00 to 0.01 | 0.01 |  |
| HighNegAff*AUC | 0.0008 | -0.01 to 0.01 | 0.86 | No significant interaction |
|  |  |  |  |  |
| African American Race in the Epidural Group | | |  |  |
| African American | -3.03 | -8.78 to 2.74 | 0.30 |  |
| Pain Unpleasantness AUC | 0.004 | 0.00 to 0.01 | 0.05 |  |
| AfrAmer*AUC | 0.01 | 0.00 to 0.02 | 0.04 | Significant interaction: being African American increases the effect of labor pain unpleasantness burden on six-week postpartum EPDS score |
|  |  |  |  |  |
| PCS in the Epidural Group | |  |  |  |
| PCS | 0.29 | 0.08 to 0.50 | 0.006 |  |
| Pain Unpleasantness AUC | 0.01 | 0.01 to 0.02 | 0.0001 |  |
| PCS*AUC | -0.0004 | -0.001 to 0.00 | 0.05 | No significant interaction |
|  |  |  |  |  |
| PSS in the Epidural Group | |  |  |  |
| PSS | -0.9 | -2.52 to 0.72 | 0.27 |  |
| Pain Unpleasantness AUC | 0.02 | -0.004 to 0.05 | 0.10 |  |
| PSS*AUC | -0.002 | -0.01 to 0.00 | 0.24 | No significant interaction |
|  |  |  |  |  |
| High Negative Affect in the No Epidural Group | | |  |  |
| High Neg Affect | 18.04 | 8.73 to 27.34 | 0.001 |  |
| Pain Unpleasantness AUC | 0 | -0.004 to 0.005 | 0.93 |  |
| HighNegAff*AUC | -0.03 | -0.05 to -0.01 | 0.007 | Significant interaction - having high negative affect decreases the effect of labor pain unpleasantness burden on six-week postpartum EPDS score |
|  |  |  |  | *NB: High negative affect is a significant predictor of six-week postpartum EPDS score in this group; findings suggest that high negative affect matters more than pain in this group* |
| African American Race in the No Epidural Group | | |  |  |
| African American | 16.73 | 7.55 to 25.90 | 0.002 |  |
| Pain Unpleasantness AUC | 0 | -0.005 to 0.005 | 0.93 |  |
| AfrAmer*AUC | -0.03 | -0.04 to -0.005 | 0.02 | Significant interaction - being African American decreases the effect of labor pain unpleasantness burden on six-week postpartum EPDS score |
|  |  |  |  |  |
| PCS in the No Epidural Group | |  |  |  |
| PCS | 0.23 | -11 to 0.57 | 0.17 |  |
| Pain Unpleasantness AUC | 0 | -0.01 to 0.01 | 0.85 |  |
| PCS*AUC | 0 | -0.001 to 0.001 | 0.66 | No significant interaction |
|  |  |  |  |  |
| PSS in the No Epidural Group | |  |  |  |
| PSS | 0.74 | -1.24 to 2.73 | 0.43 |  |
| Pain Unpleasantness AUC | 0 | -0.02 to 0.02 | 0.92 |  |
| PSS*AUC | 0 | -0.003 to 0.003 | 0.86 | No significant interaction |

*AUC, area under curve. PSS, perceived social support. PCS, pain catastrophizing scale. NA, negative affect. EPDS, Edinburgh postnatal depression scale. HighNegAff, high negative affect. AfrAmer, African American.*

**Supplemental Table 3. Multivariate regression analysis assessing relationship between labor and postpartum pain, and three-month EPDS score, among women planning and receiving epidural labor analgesia (n=55). Model integrated covariables of PSS, high NA, cervical exam at the time of epidural analgesia initiation, duration of labor, and race.**

| **Variable** | **R^2^** | **95% CI** | ***P-value*** |
| --- | --- | --- | --- |
| **Prenatal Pain Variables** |  |  |  |
| Pain returns after 2 hours (BPI-L) | 0.55 | -5.06 to 16.55 | 0.26 |
| **Labor Pain Variables** |  |  |  |
| Labor pain intensity burden (AUC) | 0.29 | 0.00 to 0.01 | 0.04* |
| Pain unpleasantness burden (AUC) | 0.30 | 0.00 to 0.01 | 0.03* |
| **Obstetric/Labor and Delivery Variables** |  |  |  |
| Sulcus Lacerations | 0.38 | 1.83 to 8.65 | 0.004* |
| Mode of Delivery | | | |
| Spontaneous vaginal delivery | 0.24 | -3.78 to 1.32 | 0.34 |
| Assisted vaginal delivery | 0.25 | -2.35 to 9.70 | 0.23 |
| Cesarean – non-reassuring fetus | 0.23 | -2.87 to 7.37 | 0.38 |
| Cesarean - arrest of descent | 0.22 | -3.17 to 3.78 | 0.86 |
| Cesarean - other | 0.22 | -6.44 to 4.99 | 0.80 |
| **Pain Variables: 6 Weeks Postpartum** |  |  |  |
| Pain right now (BPI-S #6) | 0.35 | 0.13 to 1.23 | 0.02 |
| Pain at worst past 24 hours (BPI-S #3) | 0.32 | -0.01 to 1.00 | 0.05 |
| Pain interference (BPI-S #9) |  |  |  |
| general activity | 0.40 | 0.30 to 1.40 | 0.003 |
| mood | 0.35 | 0.10 to 1.31 | 0.02 |
| walking ability | 0.38 | 0.23 to 1.22 | 0.005 |
| normal work | 0.39 | 0.27 to 1.51 | 0.006 |
| relationships | 0.41 | 0.38 to 1.66 | 0.003 |
| sleep | 0.37 | 0.18 to 1.16 | 0.01 |
| enjoyment of life | 0.46 | 0.41 to 1.31 | 0.0004 |
| **Pain Variables: 3 Months Postpartum** |  |  |  |
| Pain at worst past 24 hours (BPI-S #3) | 0.26 | -0.33 to 0.80 | 0.40 |
| Pain interference (BPI-S #9) |  |  |  |
| walking ability | 0.28 | -0.23 to 0.92 | 0.24 |
|  |  |  |  |
| *PSS, perceived social support. NA, negative affect. AUC, area under curve. BPI-S, brief pain inventory short form. BPI-L, brief pain inventory long form. CI, confidence interval.* | | | |
