# Supplementary material for: Obstetric pain correlates with postpartum depression symptoms: a pilot prospective observational study
Source: BMC Pregnancy Childbirth. 2020 Apr 22;20:240. doi: 10.1186/s12884-020-02943-7 (PMC7178606; doi:10.1186/s12884-020-02943-7)
Supplement: Supplementary file 2 — Additional file 2: Supplemental flow diagram. This diagram shows the specific variables assessed at each specific timepoint. [file 12884_2020_2943_MOESM2_ESM.pdf]

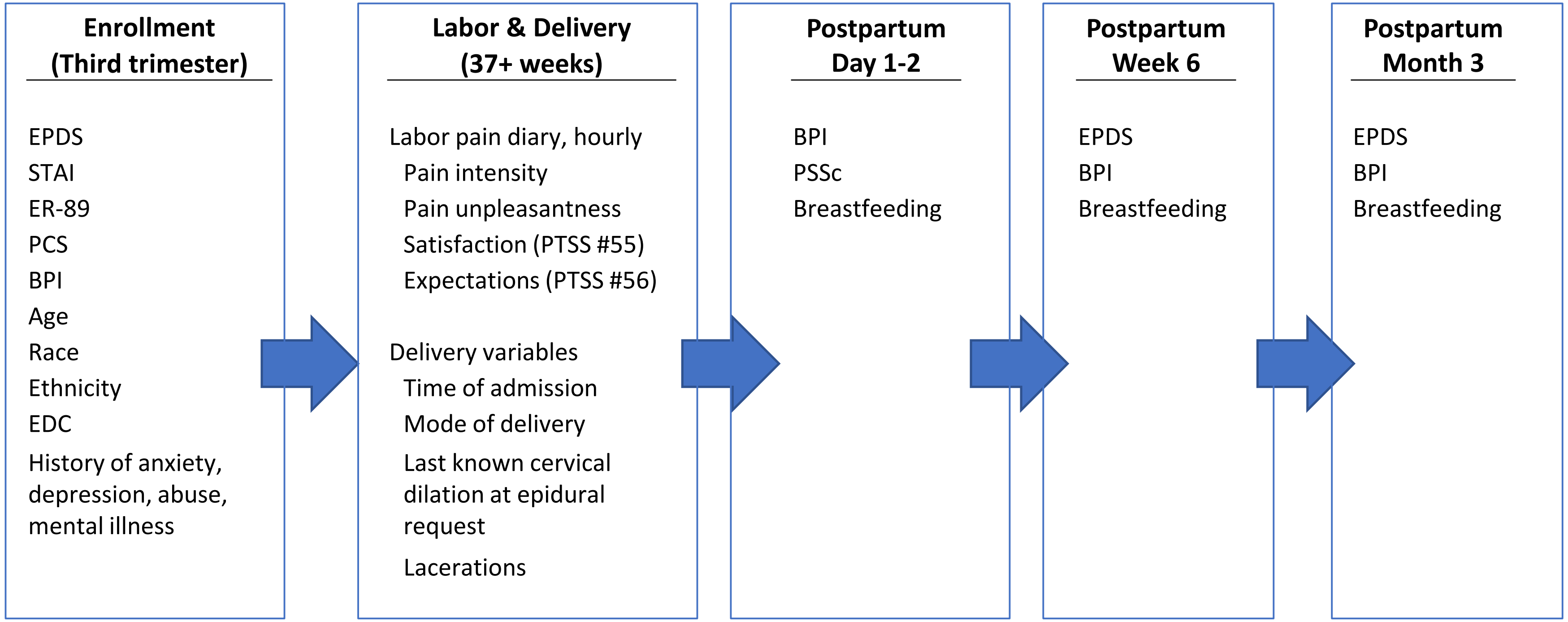

EPDS: Edinburgh postnatal depression scale; STAI, state trait anxiety inventory; ER-89, ego-resiliency scale; PCS, pain catastrophizing scale; BPI, brief pain inventory; EDC, estimated date of confinement; PTSS, Pain treatment satisfaction scale; PSSc, perceived stress scale
